# Climate and soil properties limit the positive effects of land use reversion on carbon storage in Eastern Australia

**DOI:** 10.1038/srep17866

**Published:** 2015-12-07

**Authors:** S.M.F. Rabbi, Matthew Tighe, Manuel Delgado-Baquerizo, Annette Cowie, Fiona Robertson, Ram Dalal, Kathryn Page, Doug Crawford, Brian R. Wilson, Graeme Schwenke, Malem Mcleod, Warwick Badgery, Yash P. Dang, Mike Bell, Garry O’Leary, De Li Liu, Jeff Baldock

**Affiliations:** 1 School of Environmental and Rural Science, University of New England (UNE), Armidale, NSW 2351, Australia; 2 Hawkesbury Institute for the Environment, University of Western Sydney, Richmond, NSW 2753, Australia; 3 NSW Department of Primary Industries/ UNE, Armidale, NSW 2351, Australia; 4 Department of Economic Development, Jobs, Transport and Resources, 915 Mt Napier Rd, Hamilton, Vic 3300, Australia; 5 Department of Science, Information Technology and Innovation, Dutton Park, Qld 4102, Australia; 6 Department of Economic Development, Jobs, Transport and Resources, 1301 Hazeldean Rd, Ellinbank, Vic 3821, Australia; 7 NSW Office of Environment and Heritage, PO Box U221, Armidale, NSW 2351, Australia; 8 NSW Department of Primary Industries, Tamworth, NSW 2340, Australia; 9 NSW Department of Primary Industries, Orange Agricultural Institute, Orange, NSW 2800, Australia; 10 Department of Science, Information Technology, Innovation and the Arts, Toowoomba Qld 4350, Australia; 11 Queensland Alliance for Agriculture and Food Innovation, University of Queensland, Kingaroy, Qld 4610, Australia; 12 Department of Economic Development, Jobs, Transport and Resources, 110 Natimuk Road, Horsham, Vic 3400, Australia; 13 NSW Department of Primary Industries, Wagga Wagga Agricultural Institute, PMB, Wagga Wagga 2650, Australia; 14 CSIRO Land and Water/Sustainable Agriculture Flagship, Glen Osmond, SA 5064, Australia

## Abstract

Australia’s “Direct Action” climate change policy relies on purchasing greenhouse gas abatement from projects undertaking approved abatement activities. Management of soil organic carbon (SOC) in agricultural soils is an approved activity, based on the expectation that land use change can deliver significant changes in SOC. However, there are concerns that climate, topography and soil texture will limit changes in SOC stocks. This work analyses data from 1482 sites surveyed across the major agricultural regions of Eastern Australia to determine the relative importance of land use vs. other drivers of SOC. Variation in land use explained only 1.4% of the total variation in SOC, with aridity and soil texture the main regulators of SOC stock under different land uses. Results suggest the greatest potential for increasing SOC stocks in Eastern Australian agricultural regions lies in converting from cropping to pasture on heavy textured soils in the humid regions.

Australia’s efforts to curb greenhouse gas emissions, under the “Direct Action” climate change policy, rely on the Emissions Reduction Fund, which purchases abatement (carbon credits) from project proponents undertaking approved abatement activities. Management of carbon in agricultural soils is an approved activity that generates carbon credits from conversion of cropped land to pasture, or changed management of grazing land^1^,^2^. However, evidence suggests that factors other than land use and management, such as climate and soil texture, may have a much stronger influence on soil carbon stocks^3^, limiting the positive effects of land use reversion on carbon storage.

In Australia, both pastures and adoption of conservation tillage in cropping systems (i.e. reduced or no tillage with residue retention), are estimated to sequester on average about 140 kg carbon ha^−1^ year^−1^ in the surface 10 cm soil, but climatic and edaphic constraints of the Australian environment lead to wide spatial and temporal variability in the rate achieved, and limit the confidence that this sequestration rate will be realized at any specific location^4^.

More than 90% of continental Australia can be classified as semi-arid or arid^5^, and yet the major agro-ecological regions of Australia vary from arid interiors to wet coasts with a mean annual rainfall ranging from <200 mm to >1000 mm^6^. The high variability in climatic conditions is also reflected in the adoption of a wide range of agricultural practices with varying impact on carbon cycling in agro-ecosystems^7^. In general, it is acknowledged that the carbon sequestration potential of Australian soils is much smaller than soils in the Northern Hemisphere, because of aridity, and edaphic limitations such as fertility, and chemical (i.e. sodicity and acidity) and physical constraints^8^.

Soil carbon storage is controlled by a series of hierarchical processes, including carbon inputs and outputs (decomposition, leaching and erosion). For example, the upper limit of carbon input to the soil is determined by net primary productivity (NPP) of plants, but NPP is in turn constrained by solar radiation, climate, and limitations in soil water and nutrients^8^. The decomposition of organic matter in soil depends on the climatic conditions, and the physical, chemical and biological properties of soil^9^, as well as the properties of the organic matter itself^10^.

Research in Australia on the relationship between soil carbon stocks, land use and management has revealed that in semi-arid and sub-humid zones, inclusion of pasture in cropping systems, crop residue retention, zero tillage, and phosphorus fertilizer application in pastures, have potential to improve soil carbon stocks or slow down the rate of carbon loss^11^,^12^,^13^,^14^,^15^,^16^,^17^. Despite this fact, a large body of literature suggests that certain climatic variables such as rainfall and vapor pressure deficit may account for most of the variation in soil carbon stocks when compared with land use and management effects^3^,^14^,^18^,^19^. For instance, Hobley *et al.*^20^ and Rabbi *et al.*^3^ showed that temperature and rainfall are the most important drivers of SOC storage in surface soils of New South Wales, covering an approximate agricultural production zone of 380,000 km^2^. Thus, any positive effects from land use in carbon storage may be limited by these important climatic factors. However, despite the enormous policy and management implications, we lack empirical large scale studies evaluating the relative importance of land use, climate and soil properties on SOC storage. This knowledge is, moreover, critical to predict SOC storage under changing environments.

An appropriate assessment, that can inform national climate change policy, needs to span large environmental gradients that account for the variables of influence, and should also assess the presence of interrelationships between the multiple factors influencing SOC stocks. Herein, we evaluate the relative importance of environmental variables (i.e. climate, topographic and soil properties), land uses and management practices on SOC stocks; and explore thresholds in these environmental factors that allow us to identify the best conditions to promote carbon stock of the major agricultural production areas of eastern Australia.

## Results

### Carbon stocks under different land uses and management

**Figure 1:**
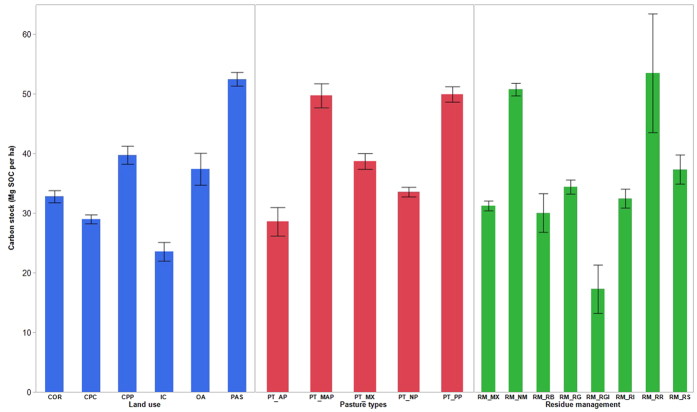
Soil carbon stock (Mg C ha^−1^) under different land uses, pasture type and residue management. (COR = Cropping, CPC = Crop/pasture (crop), CPP = Crop/pasture (CPP), IC = Irrigated cotton, OA = Organic amendments, PAS = Pasture, PT_AP = Annual pasture, PT_MAP = Mixed annual/perennial pasture, PT_MX = Mixed pasture, PT_NP = No pasture, PT_PP = Perennial pasture, RM_MX = Mixed management, RM_NM = No management, RM_RB = Residue burnt, RM_RG = Residue grazed, RM_RGI = Residue grazed and incorporated, RM_RI = Residue incorporated, RM_RR = Residue removed, RM_RS = Residue retained).

The stocks of soil carbon (0–0.3 m, adjusted for variation in bulk density) varied widely under different land uses (2–241 Mg C ha^−1^) across the 1482 sites surveyed. The SOC stock varied from 11 to 241 Mg C ha^−1^ under pasture, whereas the range was 15–43 Mg C ha^−1^ under irrigated cotton (Fig. 1). Among the crop-pasture rotations, the crop dominant rotations had lower SOC stock (2–97 Mg C ha^−1^) than pasture dominant rotations (6–166 Mg C ha^−1^). The mixed annual-perennial pasture (11–230 Mg C ha^−1^) and perennial pastures (8–241 Mg C ha^−1^) generally had the widest range and maximum average SOC stock. Similar to land uses and pasture types, the SOC stocks varied widely under different residue management regimes. The sites where no residue management was adopted appeared to have higher soil carbon stock compared to other residue management systems.

### Influence of environmental, land use and management factors on SOC stock

**Figure 2:**
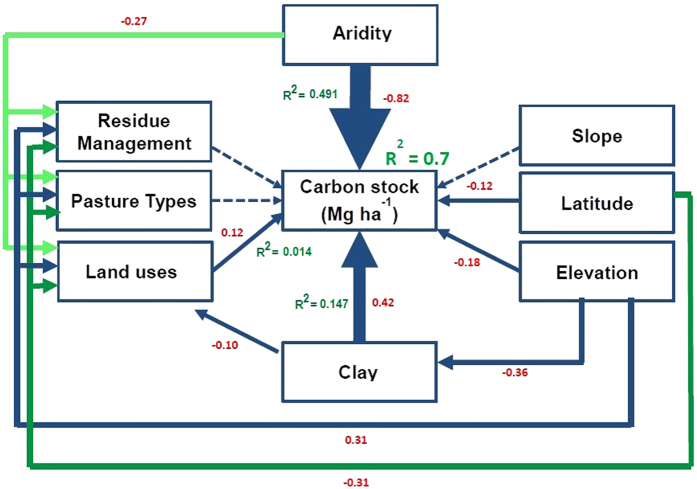
Effects of aridity, clay percentage, latitude, topographic (i.e. slope and elevation) and land uses and soil management (i.e. pasture types and residue management) variables on carbon stock of 0–30 cm soil. The model attained an acceptable fit (χ^2^ = 5.46, p = 0.141, df = 3, Bootstrap p = 0.09, RMSEA = 0.024 p = 0.913). The numbers adjacent to the arrows represent standardized path coefficients, analogous to regression weights. The width of each arrow is indicative of effect size. Continuous arrows indicate significant (p < 0.01) positive or negative relationships, whereas dashed arrows indicate non-significant relationships. The proportion of variance of carbon stock explained (R^2^) is shown above the right upper corner of the box for carbon stock. The proportion of variances of carbon stock explained by aridity, clay and land uses are shown as R^2^ adjacent to the respective arrows.

**Figure 3:**
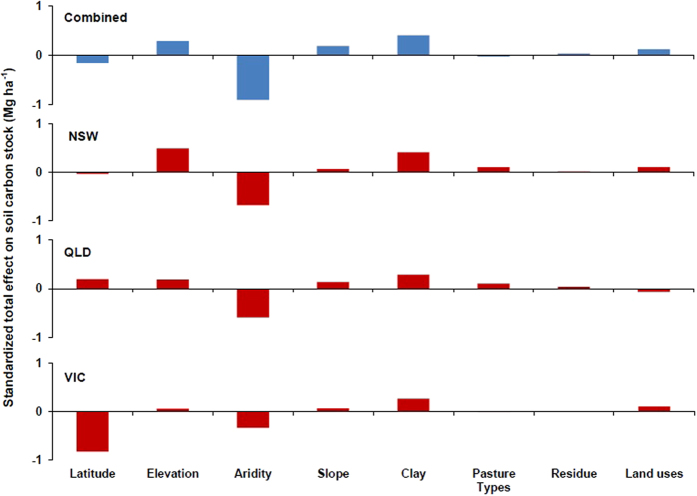
Standardized total effect (direct plus indirect) of aridity, clay percentage, latitude, topographic (i.e. slope and elevation) and land uses and soil management (i.e. pasture types and residue management) variables on carbon stock of 0–30 cm soil. The standardized total effect of each variable is shown separately for combined dataset, New South Wales (NSW), Queensland (QLD) and Victoria (VIC).

Structural equation modeling (SEM) is a simultaneous measurement method, that is, SEM measures both direct and indirect influences of environmental and management variables on SOC stocks. Exploratory factor analysis reduced the number of explanatory variables used in SEM model to aridity (i.e. integrates rainfall and potential evapotranspiration of a region^21^), latitude, slope, elevation, clay percentage of soil, land uses, pasture types and residue management. The land uses and soil management data were coded into a numerical scale to incorporate into SEM model (Table 1). Overall, climate, soil properties, land uses and management practices accounted for 70% of the total variation in SOC stock; among these variables, land uses accounted for only 1.4% of total variation (Fig. 2 and Supplementary Fig. 1). Standardized co-efficients of direct effect showed that aridity had a strong negative influence (r = −0.82, p < 0.01) on SOC stock, whereas the clay percentage of soil had a strong positive influence on SOC stock (r = 0.42, p < 0.01). Among the land use and soil management practices, land use had a significant positive (r = 0.12, p < 0.01) influence on SOC stock. However, the influence of pasture types (r = −0.021, p = 0.4) and residue management (r = 0.04, p = 0.07) on soil carbon stock were not significant. The negative relationship of carbon stock with latitude (r = −0.12, p < 0.01) and elevation (r = −0.18, p < 0.01) was significant, but the effect of slope on SOC stock was not significant (r = −0.01, p = 0.63). The standardized total effects (i.e. direct and indirect) showed that aridity and clay percentage are the most important factors determining SOC stock across Eastern Australia (Fig. 3). Furthermore, SEM model showed that aridity (r = −0.08), clay (r = −0.01), latitude (r = −0.09) and elevation (r = 0.49) had indirect effects on SOC stock through their influence on land use and soil management practices.

The SEM model was also run separately for NSW, QLD and VIC data. The SEM model showed that the standardized total effects of aridity and clay percentage on soil carbon stock were important determinants of carbon stock values in NSW, QLD and VIC (Fig. 3). The latitude had strongly negative (r = −0.84, p < 0.01) influence on soil carbon in VIC, but had had positive influence (r = 0.24, p < 0.01) on SOC stock in QLD, which may be through climatic parameters that are related with latitude (e.g. rainfall, temperature). Moreover, elevation (r = 0.20, p < 0.01) had positive influences on SOC stock in QLD.

**Figure 4:**
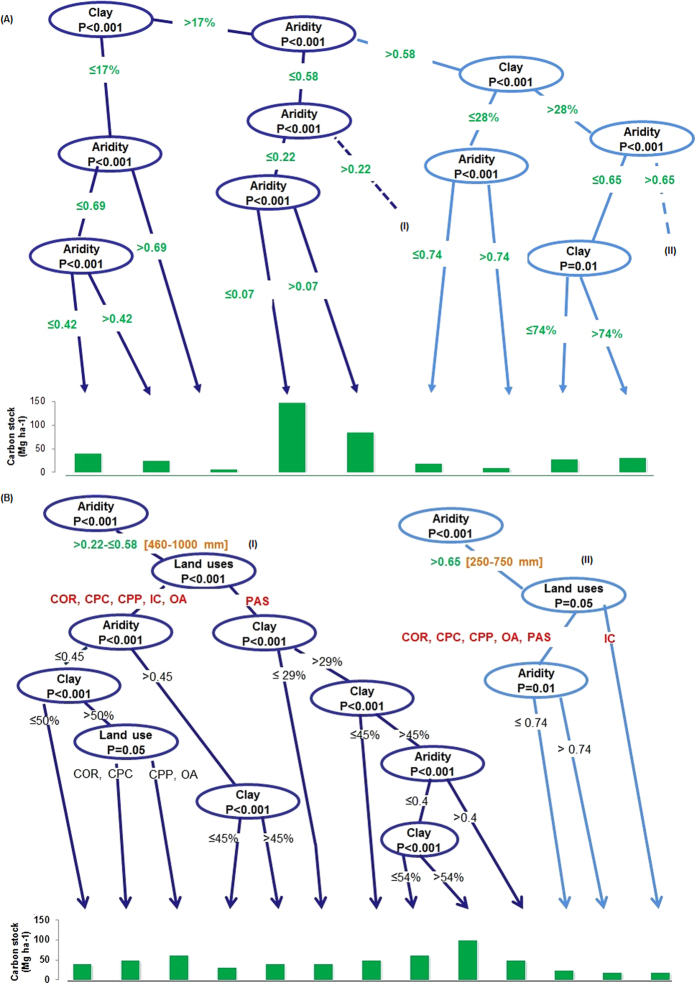
(**A**) Conditional inference tree analysis showing significant splits in aridity regions, (**B**) the aridity regions of A, with significant land use splits are indicated as I and II. The values in percent indicate percentages of clay content in soil. The bar graph represents the mean values of soil carbon stock in specific aridity and clay content. The level of significance of each split is shown inside the ovals.

The conditional inference tree (ctree) analysis further indicated that the primary patterns in SOC stock were associated with clay percentage and aridity (Fig. 4) with specific thresholds indicating significant groupings of SOC stock. In soils with low clay (<17%) there were generally low to moderate (8–41 Mg ha^−1^) amounts of organic carbon across a very wide aridity range. In these soils land use did not have a significant association with SOC stock, after clay and aridity were accounted for. In soils with higher clay (>17%) and aridity (>0.58) there was also generally low SOC stocks (ranging from 10 Mg ha^−1^ to 32 Mg ha^−1^). Within the large primary grouping of soils with >17% clay, the largest SOC stocks were associated with an aridity index ≤0.07, and secondly between 0.07 and 0.22. Land use did play a role in differentiating SOC stocks when clay was >17% but only when aridity was between 0.22 and 0.58 and >0.65.

### Effect of land uses on soil carbon stock

The conditional inference tree (ctree) analysis showed that pasture had significantly higher soil organic carbon stock compared to cropping, crop-pasture rotation, organic amendments and irrigated cotton in the regions where aridity varied between 0.22 and 0.58 (p < 0.01) (Fig. 4). Patterns existed across pasture sites that further related to soil texture and aridity: as clay increased in pasture sites, crossing thresholds of 29%, 45% and 54%, SOC stocks increased significantly. This was moderated by aridity, so SOC stocks decreased slightly in higher clay sites (>45%) when aridity increased above 0.4. At sites with aridity >0.65, irrigated cotton tended to have lower SOC stock (p < 0.05) than pasture, crop-pasture rotation and organic amendment sites, which all grouped together.

## Discussion

Our study provide strong evidence that aridity and soil properties are much more important for SOC stock than land use in Eastern Australia, one of the major agricultural production areas of Australia, suggesting that climate factor may limit any positive effects of land use reversion from cropping to pasture. These results were maintained when we repeat our analyses independently for each of three states included in this study. Interestingly, our results further suggest that the positive effects of land use on SOC stock are indirectly driven via climate and soil properties. In this respect, the effects of land use and residue management on SOC stocks are highly dependent on the climate and soil conditions at any given location. Thus, land use had an effect on soil carbon stock within specific aridity ranges, but the level of aridity and clay content of soil significantly regulated the amount of SOC present in the soil under different land uses.

The effects of spatial patterns, aridity and soil properties on soil organic carbon are well known^8^,^10^,^21^,^22^. The aridity index integrates rainfall and potential evapotranspiration of a region^23^. Air temperature, humidity, wind speed and solar radiation are the most important factors in calculating potential evapotranspiration of an area^24^. Thus, the aridity index is a quantitative amalgamation of several climatic variables, but in the Australian context, this index is very strongly related to rainfall and temperature. Intuitively, aridity of a region decreases with increasing mean annual rainfall and decreasing temperature. Since aridity is linked to these two vital climatic determinants of net primary productivity of plants, aridity influences carbon input into the soil through regulating the production of above and below ground plant biomass, water infiltration into soil, microbial processes and thus biogeochemical cycling of nutrients^8^,^21^,^22^. However, within specific aridity zones, there appears to be a hierarchy of other constraints with the next most influential being soil texture. Clay particles are involved in the formation of soil aggregates while also having large specific surface area to adsorb organic constituents and nutrient ions. In addition, clay-organic matter complexes are protected from microbial decomposition^10^, which explains the higher soil carbon stock in soils with high clay content. Previous studies have also suggested that increasing elevation (less aridity) promote SOC stock^3^. However, the current study showed lower SOC stocks under different land uses in high elevation sites compared to that of in low elevation sites. Despite the less aridity, the SOC stock was low in high elevation sites, which may be attributed to low soil productivity (e.g. soil acidity, shallow soil depth) and low air temperature. Further research is needed to examine the processes involved in carbon cycling in high elevation agricultural systems. Moreover, latitude had an overall negative impact on soil carbon stock, which was also a manifestation of change in climatic conditions with latitude. Among the three states, VIC has the sharpest increase in aridity with increasing latitude. As the SEM estimates are based on partial correlation, the standardized direct negative effect of latitude, especially in VIC, might indicate the unaccounted influence of soil types, aspect of slope and management practices on the carbon stock, similar to recent SEM approaches using spatial coordinates^21^.

Although the contribution of land use to variation in SOC stock observed in this study was small (1.4% of total explained variation), the relationship between SOC stock and land uses showed that pasture and pasture dominated crop-pasture rotation do have potential to store higher organic carbon than systems dominated by cropping. Data from Australian long term experimental sites have shown that land use change from continuous cropping to pasture or crop-pasture rotation could slow down the rate of decline in SOC stocks or increase depending on the initial SOC level^11^,^12^. This was reflected in the results of this study, particularly in the ‘moderate’ zone of aridity. In the current study, the effect of pasture land use on SOC stock was very pronounced in the aridity index range of 0.22 to 0.58, which corresponds approximately to the 460–1000 mm annual rainfall zone. In this range pasture had higher carbon stock than cropping, crop-pasture rotation, irrigated cropping and organic amendment sites. These pasture sites had not been mechanically disturbed for at least 10 years. This would have promoted formation of mechanically stable macro- and micro-aggregates in soil^25^. Moreover, plant roots contain higher lignin and other recalcitrant macromolecules including suberin, which reduce the decomposition rate of roots in soil compared to shoots^26^. High amounts of such recalcitrant carbon in the pasture sites would also help account for this pronounced difference within this aridity range.

The low organic carbon stock under all land uses at high aridity compared with low and moderate aridity reflects the lower capacity of NPP in high aridity zones to contribute below ground organic input to build SOC stock. This is evidence of an overarching abiotically controlled limitation to SOC accrual in these systems. Despite this, there is some evidence for increases in SOC stock to be made in these zones through land use change. The pasture, cropping and crop-pasture land uses had significantly higher carbon stocks than irrigated cotton. Irrigated cotton sites were intensively cropped, with cotton most often grown in rotation with wheat and short-duration legumes^27^ but with mainly conventional tillage practices. The deterioration of soil structure by conventional tillage and application of chemical fertilizers to supplement nitrogen and phosphorus in soil, as well as irrigation, would increase carbon turnover. At aridity levels <0.22, carbon stocks in different land uses were statistically similar. In this low aridity zone, land use was mainly pasture and the carbon input is most likely both high and consistent due to adequate moisture availability and favourable temperature for pasture growth. Land use did not have any influence on carbon stocks in soil when aridity was between 0.58 and 0.65. The absence of land use effect on carbon stock in this aridity range suggests that the soils in these sites may have reached, or be close to, their maximum capacity to store carbon in this environment where carbon stocks are naturally limited due to low rainfall coupled with high temperature. This interpretation is mainly based on the limited range of land uses and broad scale of characterization examined in the current study. That is, there may be greater probability for organic carbon stock increase in pastures of greater than 10 years duration, but further research is required to assess this. This would require detailed examination of long term trial sites in conjunction with likely aridity thresholds. Appropriate modelling of organic carbon interaction with land uses across different climatic zones would also be a feasible approach for examining this.

### Conclusion

By examining a very large and unique dataset of land use and carbon measurements across eastern Australia (1482 sites) we found that the differences in land use and management practice explained only 1.4% of total variation in carbon stocks across the whole data set, while climatic and soil related variables explained 64%. The effect of land use on organic carbon stocks was strongly regulated by aridity and clay content of soil. This suggests that any attempt to influence soil organic carbon stock through change in land use and soil management practices needs to consider the overriding influence of the local environment, as well as the carbon sequestration potential of a soil in a specific climatic situation.

## Methods and Materials

### Site selection

**Figure 5:**
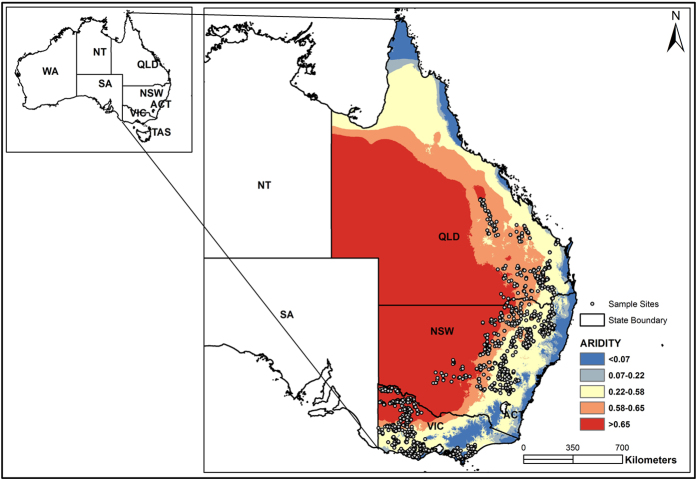
Sample sites and aridity thresholds in New South Wales (NSW), Queensland (QLD) and Victoria (VIC) [Created with ArcGIS 10.2].

Under the National Soil Carbon Research Program (SCaRP), soil under different agricultural management practices in New South Wales (NSW), Queensland (QLD) and Victoria (VIC), Eastern Australia, was sampled and soil organic carbon (SOC) was quantified. A survey approach was employed, which sampled 1482 sites, extended over approximately 830,900 km^2^, covering the major land uses and soil management systems in the three states (Fig. 5). Further detail on site selection and rationale can be found in Cowie *et al.*^28^.

### Sampling

A 25 × 25 m quadrat was established at each sampling location, and the GPS coordinates (WGS 84) recorded in the southwest corner. When sampling cropping sites, the grid was oriented 30^o^ to the crop row to prevent sampling bias with respect to the row and inter-row area. Ten sampling points were located using random coordinates within the quadrat. Soil cores were collected using a manual soil corer (metal tube 50–70 mm in diameter). Surface litter was removed prior to coring. Intact cores were extracted to a depth of 0.3 m, separated into 0.1 m depth increments^29^.

### Site history

Management data for the 10 years prior to the inception of soil sampling in 2009, were collected by a survey with landholders, as outlined by Sanderman *et al.*^29^. The survey of land management practice covered land uses, tillage practice, fallowing, residue management, pasture type, grazing management, irrigation and soil conditioners (i.e. agricultural lime, gypsum). The land uses and soil management practices are herein called management practices.

### Climate and topographic data

The aridity index data was extracted from the Consortium for Spatial Information (CGIAR-CSI) (http://www.cgiar-csi.org/data/global-aridity-and-pet-database) using the latitude and longitude of each specific sampling site. The aridity index was modelled by CGIAR-CSI using very high resolution (~1 km) WorldClim global climate data (http://worldclim.org/)^21^,^22^,^30^,^31^. We used aridity (1-Aridity Index) as a surrogate for rainfall, temperature, vapor pressure deficit of the sampling sites, as undertaken in recent similar work^30^. The elevation (m) and slope (percent), of each site were extracted from the Smoothed Digital Elevation Model of Australia (DEM-S), which was derived from the 1 second resolution SRTM data acquired by NASA in February 2000. The climate and topographic data are considered henceforth as environmental variables.

### Sample processing

A composite sample for each depth was created by combining the soil from each depth increment across the ten cores collected from each site. Soil samples were air-dried at 40 °C until constant weight. The sample processing, including bulk density measurement was as detailed in Cowie *et al.*^28^.

### Soil analyses

Baldock *et al.*^32^ provides details of the methodology for analyses of total organic carbon (SOC) in soil. Total soil carbon stocks were expressed as total organic carbon to 0.3 m depth in Mg ha^−1^. To account for possible impacts of management on bulk density, stock of SOC, was expressed on the basis of equivalent soil mass^3^. The gravimetric content of clay, soil pH and concentrations of Si and Al were determined by applying predictive algorithms developed by Janik *et al.*^33^ and Janik and Skjemstad^34^ to the mid-infrared (MIR) spectra acquired for all samples^3^.

### Data coding

Land use and soil management history for 10 years prior to sampling were recorded detailing tillage type, residue management, grazing management, pasture type, irrigation, use of soil conditioners and use of a long fallow period for each sampling site. The categorical data of land use and management practices were then converted into a set of dummy variables (1–10) as per Rabbi *et al.*^3^ (Table 1). Based on the *a priori* understanding of the effect of land uses and management on soil carbon stock^3^, the values between 1 and 10 were assigned to land uses and management classes in the order of their increasing positive influence on soil carbon stock. The sites that were under continuous pasture or continuous cropping for the 10 years were coded as pasture or cropping, respectively. Land uses were coded as crop dominant and pasture dominant crop/pasture sites depending on the numbers of years under crop or pasture in 10 year period. Sites with application of organic amendments, and irrigated cotton were coded separately. Among the sampling sites a subset of cropped sites was irrigated and received soil conditioners. Long fallow was not common, but a subset of crop/pasture rotation sites included this practice.

### Statistical Analyses

We used structural equation modeling (SEM) to analyze the multivariate relationships between explanatory variables (i.e. environmental, land uses and management practices variables) and soil carbon stocks. Structural equation modeling analyzes a system of pre-specified linkages between causes and effects, and assigns explanatory power (or explainable variation) to the links between variables. Partial correlations as well as relationships between explanatory variables can be analyzed by SEM, which are both essential in estimating the effect size associated with dependent variables. The capacity of SEM to separate direct and indirect effects of a variable on dependent variables is considered one of the most important advantages of SEM^35^.

Before performing SEM, multiple regression and exploratory factor analysis were performed to check the collinearity between environmental, soil, land use and management variables and to reduce the number of variables selected for SEM. Multiple regressions were performed using SPSS 22 (IBM, New York, USA). The variables that had variance inflation factor (VIF) ≥3.0 were considered to have collinearity^36^. Due to the high VIF between bulk density, soil pH, clay and Fe, clay was retained for SEM. Aridity, elevation, slope and latitude data had VIF <3 and retained for factor analysis. Since the VIFs of tillage and land use were >3.0 (with the intensity of cropping being strongly related to tillage intensity), tillage was not used as a management variable. Following these collinearity checks, an exploratory factor analysis was performed only with the variables that had VIF ≤3 to further reduce the number of variables. Aridity, elevation, slope and latitude data had factor loadings between 0.4 and 0.8, so retained for SEM. Among the variables considered as relating to management practices, land use, pasture type and residue management had factor loadings 0.6–0.8, allowing all three to be retained in the SEM.

We performed structural equation modeling using AMOS 21 (IBM SPSS, Amos Development Corporation, Meadville, Pennsylvania, USA) to evaluate the influence of environmental variables (i.e. latitude, aridity, elevation and slope), soil properties (i.e. clay) and soil management practices (i.e. land use, pasture types, residue management) on soil carbon stocks of Eastern Australia. To construct a SEM we hypothesized that the soil carbon stock was dependent on the land uses, management practices, soil properties and environmental variables. The clay percentage was dependent on latitude, aridity, elevation and slope. Moreover, the management practices were also dependent on the latitude, aridity, clay, slope and elevation of each site. Since land uses and management data were converted to a set of dummy variables (1–10), these categorical variables were used as ordinal data in SEM. The number of classes in land uses and management practices were ≥5, is deemed the cut-off for a reliable SEM estimate with ordinal data^37^. Moreover, and as an additional check on the reliability of the use of these dummy variables, we also compared the maximum likelihood SEM estimates with Bayesian estimates of SEM and found that the presence of ordinal variables did not have any influence on the SEM estimates, as per recommended procedure^37^.

Non-significant chi-square (χ^2^) test, goodness of fit index (GFI) and root mean square error of approximation (RMSEA) were used to find an acceptable SEM model^38^,^39^. Additionally, we confirmed the fit of the model using the Bollen–Stine bootstrap test^21^. Our a-priori model attained an acceptable fit by all criteria, with few alterations. The initial SEM model was accepted after setting co-variances between land use, pasture types and residue management. We also allowed elevation and slope to co-vary with latitude. The accepted SEM model for combined data was tested separately with NSW, QLD and VIC data and attained an acceptable fit without alteration. The standardized estimates (i.e. total and direct effects) of the regression weights and the coefficient of determination were used to describe the relationship between carbon stocks, environmental variables, land uses and management practices.

We utilized the accepted SEM model to identify environmental and management variables that had a strong influence on soil carbon stock. It is also implied in the SEM model that management variables had a significant effect on soil carbon stock under specific sets of the environment variables. A conditional inference tree (ctree) analysis was performed to identify thresholds of effects of the selected explanatory variables from SEM analysis (i.e. aridity, clay content and land uses) on soil carbon stock. Conditional inference trees estimate a regression relationship by testing global null hypothesis of independence between any of the input variables and the response. The estimation stops if the null hypothesis of independence cannot be rejected. Otherwise the input variable with strongest association to the response is selected. The ctree analysis then recursively implements binary splits in the selected input variables. Conditional inference tree analysis is essentially a pattern recognition procedure, and as such can determine significance of patterns within existing datasets^40^. However, the method is dependent on having at least several data points within the different potential defined categories and can only model variability within the provided data^40^,^41^. Because of this, and the nature of the survey, the land uses and management combinations surveyed in different soils and climatic conditions were significantly unbalanced, so separately analyzing associations after breaking the dataset into different land uses may have led to significant increases in errors^3^. The ctree analysis was performed using “partykit” package in R^41^.

## Additional Information

**How to cite this article**: Rabbi, S.M.F. *et al.* Climate and soil properties limit the positive effects of land use reversion on carbon storage in Eastern Australia. *Sci. Rep.* **5**, 17866; doi: 10.1038/srep17866 (2015).

## Supplementary Material

Supplementary Information

## Figures and Tables

**Table 1:** Definitions of land uses and management practices.

Management	Definition	Abbreviation	Numeric code
Land use			
Cropping	Cropped only in last 10 years	COR	1
Irrigated cotton	Cotton mostly in rotation with cereal/grain legume/sorghum in last 10 years. Some sites have no- crop between cotton rotations.	IC	2
Crop/pasture (crop)	Last 10 years include 5–9 years of cropping with pasture in remaining years (crop dominant). This category also includes the sites that were under cropping for 5 years and with 5 years under pasture.	CPC	3
Crop/pasture (pasture)	Last 10 years include 5–9 years of pasture with cropping in remaining years (pasture dominant).	CPP	4
Organic amendments on cropping and pasture	Crop, pasture, crop/pasture (crop) and crop/pasture (pasture) sites that received organic amendments	OA	5
Pasture	Pasture only in last 10 years	PAS	6
**Tillage**			
Conventional Tillage	Cultivation of soil, use of disc or tine implements for soil preparation and weed control	Til_CT	1
Mixed Tillage	More than one type of tillage practiced in last 10 years	Til_MX	2
Minimum Tillage	Minimum tillage, weeds were mainly controlled by herbicides	Til_MT	3
Zero Tillage	Crops are sown by direct-drilling, weeds are mainly controlled by herbicides	Til_ZT	4
No soil disturbance	Pasture sites, no working of soil	Til_ND	5
**Residue**			
Residue baled or removed	Residue baled or removed (organic amendment and crop/pasture (crop) sites)	RM_RR	1
Residue incorporated	Residue incorporated in to soil using conventional tillage practices	RM_RI	2
Residue grazed and incorporated	Residue grazed and incorporated in to soil using conventional tillage practices	RM_RGI	3
Residue grazed	Residue grazed	RM_RG	4
Mixed management	More than one type of residue management practiced in last 10 years	RM_MX	5
Residue burnt	Residue burnt	RM_RB	6
Residue retained on surface	Residue retained on surface(zero and minimum tilled sites)	RM_RS	7
No management	No residue management (pasture and some of pasture or crop dominant crop/pasture sites)	RM_NM	8
**Grazing**			
No grazing	No grazing (crop dominant crop/pasture sites)	GM_NG	1
Other	Grazing other than the dominant types	GM_other	2
Set stocking	Pasture sites, continuous grazing	GM_SS	3
Rotational grazing	Pasture sites, grazing is restricted to short periods of several days, followed by long rest periods, generally of several months depending on pasture condition	GM_RG	4
Mixed set stocking and Rotational grazing	Mixture of set stocking and rotational grazing	GM_SSRR	5
Mixed grazing	More than one type of grazing management practiced in last 10 years	GM_MX	6
No grazing management	Pasture or cropped sites with no grazing history	GM_NGM	7
**Pasture Type**			
No pasture	Cropped and crop dominant crop/pasture sites	PT_NP	1
Annual pasture	Annual pasture, either grass or legume dominant	PT_AP	2
Mixed pasture	Crop dominant and pasture dominant crop/pasture sites where a crop year with ‘no pasture’ and pasture year with annual/perennial pasture	PT_MX	3
Mixed annual/perennial pasture	Mixed annual/perennial pasture, either grass or legume dominant	PT_MAP	4
Perennial pasture	Perennial pasture either grass or legume dominant	PT_PP	5
