# Supplementary material for: Climate and soil properties limit the positive effects of land use reversion on carbon storage in Eastern Australia
Source: Sci Rep. 2015 Dec 7;5:17866. doi: 10.1038/srep17866 (PMC4671085; doi:10.1038/srep17866)
Supplement: Supplementary Information [file srep17866-s1.pdf]

Supplementary Material

Climate and soil properties limit the positive effects of land use reversion on carbon storage in Eastern Australia.

S.M. Fazle Rabbi[1], Matthew Tighe[1*], Manuel Delgado-Baquerizo[2], Annette Cowie[1,3], Fiona Robertson[4], Ram Dalal[5,], Kathryn Page[5], Doug Crawford[6], Brian R Wilson[1,7], Graeme Schwenke[8], Malem Mcleod[8], Warwick Badgery[9], Yash Dang[10], Mike Bell[11], Garry O'Leary[12], De Li Liu[13], Jeff Baldock[14]

[1]*School of Environmental and Rural Science, University of New England (UNE), Armidale, NSW 2351, Australia*

[2]*Hawkesbury Institute for the Environment, University of Western Sydney, Richmond, NSW 2753, Australia*

[3]*NSW Department of Primary Industries, Armidale, NSW 2351, Australia*

[4]*Department of Economic Development, Jobs, Transport and Resources, Hamilton, Vic 3300, Australia*

[5]*Department of Science, Information Technology and Innovation, Dutton Park, Qld 4102, Australia*

[6]*Department of Economic Development, Jobs, Transport and Resources, Ellinbank, Vic 3821, Australia*

[7]*NSW Office of Environment and Heritage, Armidale, NSW 2351, Australia*

[8]*NSW Department of Primary Industries, Tamworth, NSW 2340, Australia*

[9]*NSW Department of Primary Industries, Orange Agricultural Institute, Orange, NSW 2800, Australia*

[10]*University of Queensland, Toowoomba Qld 4350, Australia*

[11]*School of Agriculture and Food Science, University of Queensland, Gatton, Qld 4343, Australia*

[12]*Department of Economic Development, Jobs, Transport and Resources, Horsham, Vic 3401, Australia*

[13]*NSW Department of Primary Industries, Wagga Wagga Agricultural Institute, Wagga Wagga 2650, Australia*

[14]*CSIRO Sustainable Agriculture Flagship, Glen Osmond, SA 5064, Australia*

**Corresponding Author, mtighe2@une.edu.au*

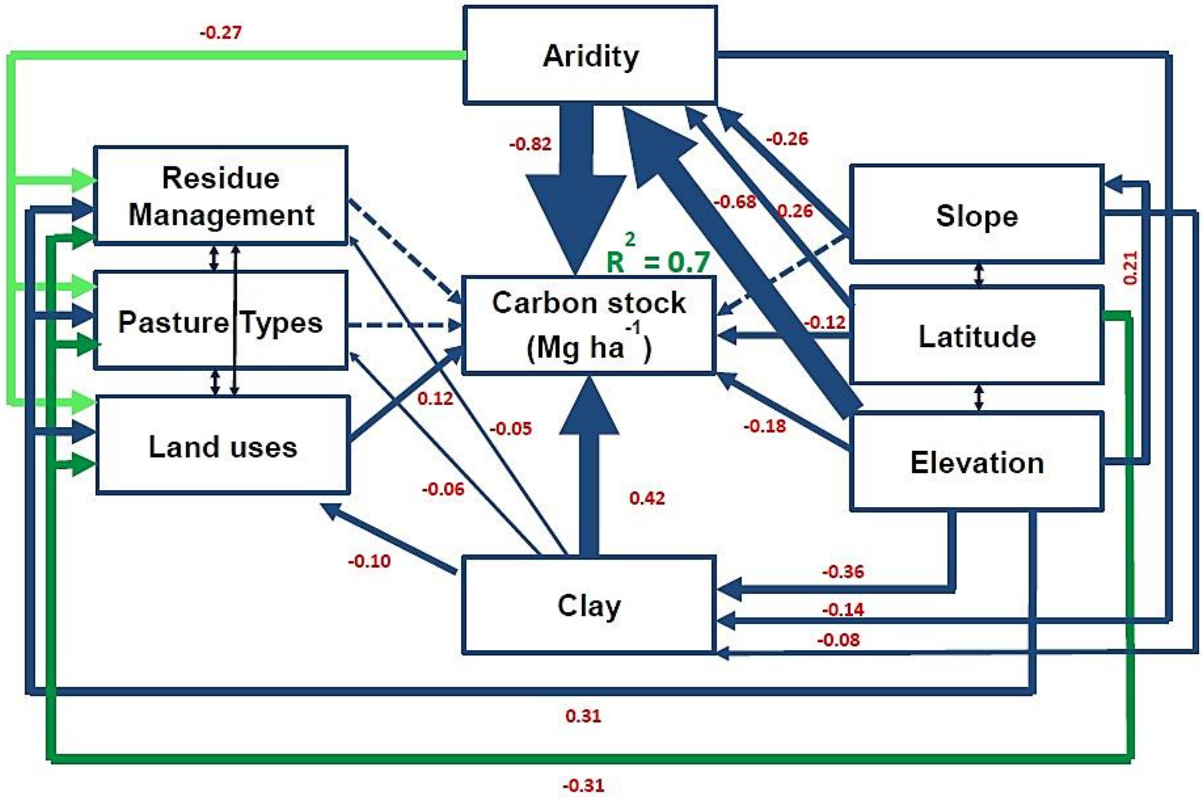

Supplementary Figure 1| Effects of aridity, clay percentage, latitude, topographic (i.e. slope and elevation) and land uses and soil management (i.e. pasture types and residue management) on carbon stock of 0-30 cm soil. The model attained an acceptable fit (χ^2 = 5.46, p = 0.141, df = 3, Bootstrap p = 0.09, RMSEA = 0.024 p = 0.913). The numbers adjacent to the arrows indicate standardized path coefficients, analogous to regression weights. The width of each arrow is indicative of effect size. Continuous arrows indicate significant (p<0.001) positive or negative relationship, whereas dashed arrows indicate insignificant relationships. The proportion of variance of carbon stock explained (R^2) is shown above the right upper corner of the box for carbon stock.
